# Optimization of pectin yield extracted from coffee Arabica pulp using response surface methodology

**DOI:** 10.1016/j.heliyon.2024.e29636

**Published:** 2024-04-12

**Authors:** Girma Biratu, Henock Woldemichael Woldemariam, Girma Gonfa

**Affiliations:** a Department of Chemical Engineering, Addis Ababa Science and Technology University, P.O. Box 16417, Addis Ababa, Ethiopia; b Biotechnology and Bioprocess Center of Excellence, Addis Ababa Science and Technology University, P.O. Box 16417, Addis Ababa, Ethiopia

**Keywords:** Coffee pulp, Pectin, Degree of acetylation, Optimization, Response surface methodology

## Abstract

Pectin was extracted from coffee pulp using 0.1 M H2SO4. The Box Behnken Design based Response surface methodology was applied to optimize pectin yield. The impact of extraction time (45–75 min), temperature (80–100 °C), solid to liquid ratio (SLR) (1:20, 1:27.5, and 1:35), and pH (1.5, 2, and 2.5) on pectin yield were studied. Under optimal extraction conditions (84 °C, 75 min, SLR of 1:20 and pH: 1.5), physical, chemical, structural and antioxidant properties of pectin were examined. The results of the physicochemical analysis are: acetyl value: 1.10 ± 0.05 %, equivalent weight: 1429 ± 54 g/mol, anhydrouronic acid: 57.1 ± 0.9 %, degree of esterification: 78.5 ± 1.8 %, moisture content: 8.5 ± 1.5 % and ash content: 4.3 ± 0.9 %. FTIR analysis indicated the (-OH) peak of pectin was lower and shifted left compared to treated and untreated coffee pulp powder. SEM analysis shows a smoother surface, whereas XRD shows a less amorphous structure of pectin. The total phenolic and flavonoid content of coffee pulp pectin was found to be 26.7 μg Gallic Acid Equivalent/mg and 0.8957 μg Quercetin Equivalent/mg, respectively. Antioxidant analysis showed significant antioxidant properties (IC50 = 642.31 ± 30.43 μg/mL). The predicted and actual pectin yields at the optimal extraction condition were 14.39 and 13.7 %, respectively, with R^2^ = 0.95 that indicate the model can represent the experiment. Therefore, achieving a maximum pectin yield with improved antioxidant and other physicochemical qualities ensures that coffee pulp can potentially serve as a viable commercial source of pectin.

## Highlights

• Pectin extraction from coffee pulp was optimized.
• Modeling of pectin yield was conducted using the Box-Behnken design.
• The optimum extraction conditions were 84 °C, 75 min, SLR of 1:20, and a pH of 1.5.
• Pectin derived from coffee pulp is classed as high methoxyl pectin.

## 1 Introduction

Pectin is one of the polysaccharides found in the cell walls of higher plants. It contains rhamnogalacturonan (I and II), homogalacturonan, and xylogalacturonan. Its galacturonic acid structure is joined through α-(1–4) glycosidic bonds. It is divided into two types: low methoxyl pectin (less than 50 % methylation) and high methoxyl pectin (more than 50 % methylation) [1]. Food industries employed pectin as a thickening, gelling, texturizing, emulsifying and stabilizing ingredient [2]. A study by Shahrin, Narudin [3] also indicated pectin can be utilized as an adsorbent that can remove toxic substances as a result of its excessive hydroxyl group. Pectin application in pharmaceutical, biotechnologies, food, beverage and packaging industries increases pectin market globally [4]. For example, the worldwide pectin market reached 838,600 tons in 2022 and is predicted to rise at a CAGR of 3.1 % until 2028 [5]. This increment in pectin demand and citrus fruit diseases challenged the balance of market demand and supply of pectin [6]. Numerous studies have been conducted over the last two decades to extract pectin from agro-industrial wastes such as cranberries [7], onions [8], garlic [9], banana [10], mangoes [11], pumpkins, peaches, rapeseed, papaya [12], citrus maxima [13] and others. Still, an extensive works on pectin extraction from some other by products such as coffee wastes requires more attention. In comparison to commercial pectin, pectin from by-product contains more antioxidants, colourants, and other bioactive substances [14].

Coffee is one of an extensively farmed commercial crop in the world. In 2022 alone, world coffee production was 10.37 million ton [15]. During the wet processing of coffee, waste generated is as much higher as about 50 % of the coffee cherries [16,17]. The skin and pulp, collectively known as coffee pulp, represent 40–50 % mass of coffee cherries [18]. Coffee pulp has significant pectin content up to 15 % based on dry bases [1,19].

Commercial pectin production uses acid extraction since acids are the most effective extracting agents [19]. Industrial pectin is extracted using solutions of nitric, sulfuric, hydrochloric, and organic acids at pH 1–3 and temperatures of 70–90 °C for 1–12 h [20]. Pectin extraction entails preparing an alcohol-insoluble residue (AIR), extracting pectin from the alcohol-insoluble residue, isolating, purifying, and drying the recovered pectin. Pectin yield and properties depend on the raw materials and extraction procedures used to extract pectin. A few researchers extracted pectin from coffee pulp and characterized it [19,[21], [22], [23], [24]]. To the best of our knowledge, none of them optimized pectin yield using Response Surface Methodology (RSM). RSM is a set of statistical tools for process design and optimization. It is used to optimize process where multiple factors and potential interactions may affect the result [25]. Pectin from Sunflower heads [26], orange peels [27], and melon peels [28] were some works that used RSM for optimization of pectin yield. The purpose of the study was to use response surface methodology to extract coffee pulp pectin with the highest possible yield and desired quality. In this study, pectin was extracted using hot acidified water (0.1 M sulfuric acid solution). The study also attempted to characterise the purity and quality of extracted pectin using degree of esterification, methoxyl content, moisture and ash content, degree of acetylation, anhydrouronic acid, morphological and structural analysis, total phenolic and flavonoid content and antioxidant activity.

## 2 Materials and methods

### 2.1 Materials

Coffee cherry (Harar coffee) was harvested on 9–13/11/22 from the Mechara Agricultural Research Centre, West Hararghe, Oromia, Ethiopia. The coffee cherry was washed under cold water, packaged, and kept at 4 °C.

### 2.2 Coffee pulp preparation

The sample preparation method was adopted from Reichembach and de Oliveira Petkowicz [19] with some modifications. The coffee cherry was sorted, manually depulped, and immediately frozen in a deep freezer (Thermo Scientific Forma 88000 Series 86 °C Freezers). The coffee pulp was then ground using an electronic grinder (NM-8300, Japan) after being oven dried at 45 °C for 24 h. Following that, 100 g of coffee pulp powder was heated for 25 min in 670 mL of 96 % (v/v) ethanol (ISO LAB chemicals) to obtain alcohol-insoluble residue. Finally, the alcohol-insoluble residue was filtered, rinsed three times with 100 mL ethanol (absolute), dried at ambient temperature for 10 h, milled, and kept at 4 °C until further use. All chemicals used in these experiments are analytical grade.

### 2.3 Pectin extraction from coffee pulp

Pectin was extracted from alcohol insoluble residue (AIR) using 0.1 M H_2_SO_4_ at pH of 1.5, 2.0, and 2.5 (adjusted by addition of 0.1 M NaOH), temperatures of 80, 90, and 100 °C, time of 45, 60, and 75 min and SLR of 1:20, 1:27.5, and 1:35 (wt./v) extraction conditions. The filtrate was separated from residue with a double muslin cloth and precipitated with 95 % ethanol that was twice the volume of extract and put at 4 °C for 16 h. The precipitate was then filtered, with 100 mL of ethanol (absolute), and air dried, giving rise to Coffee pulp pectin (CPP). Then, the yield of CPP was calculated using equation (1).

(1) $$Y\left(\%\right)=\frac{{W_{t}}_{2}}{{W_{t}}_{1}}\times 100$$

where, ${W_{t}}_{1}$ weight of alcohol treated coffee pulp powder and ${W_{t}}_{2}$ is weight of dried pectin.

### 2.4 Characterization of pectin

#### 2.4.1 Acetyl value of pectin

The acetyl value was determined following the procedure of Virk and Sogi [29] with some modifications. Pectin (0.5 g) dissolved in 0.1 M NaOH solution (25 mL) and put overnight. A prepared pectin solution was diluted with distilled water to 250 mL from which 20 mL was transferred to the vacuum rotary evaporator. Next, a 20 mL magnesium sulfate-sulfuric acid solution (prepared by mixing magnesium sulfate (100 g) with sulfuric acid (98 %) (1.5 g) in 20 mL of distilled water) was diluted to 180 mL. Following that, the solution transferred to a vacuum rotary evaporator with 20 mL of sample aliquot until all the distillate was recovered. A blank solution of magnesium sulfate-sulfuric acid with equal volume was also prepared and distilled. Moreover, the distillates were titrated against 0.05 M NaOH using a phenolphthalein. Finally, the Acetyl value was calculated by using equation (2).

(2) $$\text{Av}\;\left(\%\right)=\frac{V_{s}-V_{b}\;}{W_{t}\;}\times N\;\text{of}\;\text{NaOH}\times 4.3$$

where, *V*_*s*_ is volume of sample, *V*_*b*_ is volume blank expressed in mL, *N* is Normality of a NaOH, *W*_*t*_ is weight of the sample and 4.3 are tenth of molecular weight of acetyl.

#### 2.4.2 Methoxyl content (MeO)

MeO determination was conducted with method described by Patra and Basak [30]. The neutral solution from the Ew analysis was taken and 0.25 N NaOH (25 mL) was added, shaken and stood for 30 min in a stoppered flask at ambient temperature. Then, 0.25 M HCl (25 mL) was added. Finally, titration was conducted using 0.1 M NaOH until a pink colour is obtained. Finally, MeO was determined using equation (3).

(3) $$\text{MeO}=\frac{\left(V\times M\right)\;\text{of}\;\text{alkali}\times 3.1}{W_{t}\;}$$

where, *W*_*t*_ is of sample weight (g), *M* is molarity of alkali*.* and *V* is volume of alkali (mL).

#### 2.4.3 Anhydrouronic acid (AUA)

The AUA content of coffee pulp pectin was obtained using equation (4) [31]:

(4) $$\text{AUA}\left(\%\right)=\frac{176\times 0.1\left(z+y\right)}{W_{t}\times 1000}\times 100$$

where, 176 is a molecular unit of AUA, y is mL of NaOH from MeO determination, *W*_*t*_ is sample weight, and z is mL of NaOH from Ew determination.

#### 2.4.4 Degree of esterification (DE)

DE of coffee pulp pectin was determined using equation (5) [31].

(5) $$\text{DE}\;\left(\%\right)=\frac{\text{MeO}\;\left(\%\right)\times 176}{\text{AUA}\;\left(\%\right)\times 31}\times 100$$

where, *MeO* is methoxyl content, *AUA* is anhydrouronic acid, and *31* is methoxyl molecular weight.

#### 2.4.5 Equivalent weight (Ew)

A pectin equivalent weight was dealt based on a method reported by Patra and Basak [30]. A 0.1 g of CPP was first mixed with 5.0 mL ethanol and 1.0 g of sodium chloride before being diluted to 100 mL with distilled water. To undergo a titration, six drops of phenolphthalein was added to the pectin solution and then after a 0.1 N NaOH was added until end point. Then, equivalent weight of was calculated using equation (6).

(6) $$\text{Ew}=\frac{W_{t}\;}{V\times N}\times 1000$$

where, *Ew* is equivalent weight (g/mol), *N* is Normality of alkali, *Wt.* is weight of sample (g) and *V* is volume of NaOH (mL).

#### 2.4.6 Ash and moisture content

The ash and moisture content of coffee pulp pectin were tested in accordance with AOAC [32].

#### 2.4.7 Fourier transform infrared spectroscopy (FTIR) analysis

Coffee pulp powder, treated coffee pulp powder and coffee pulp pectin were analyzed according to Pereira, Vieira [33] using Fourier Transform Infrared Spectroscope (iS50ABX, USA). After pressing 2 mg of ground sample into thin slices, the FTIR spectrum of sample was collected on a FTIR spectrophotometer from 400 to 4000 cm^−1^.

#### 2.4.8 SEM analysis

The morphology of the coffee pulp pectin and powder was studied using Scanning electron microscope (LEO 1450 VP, England) according to Pereira, Vieira [33]. Samples were covered with 2 nm of Au to improve conductivity. After that, aluminium pin stub was placed inside a Phenom Standard Sample Holder (SH) and tested with 10 kV voltage and magnification of × 1500.

#### 2.4.9 XRD analysis

The analysis of X-ray diffraction was conducted according to Kazemi, Khodaiyan [34]. Recording of patterns was conducted using an X-ray diffractometer (D8-Advanced, Bruker, Karlsruhe, Germany) Cu K-alpha radiation (1.5406 Å), 10–80° diffraction angle (2θ), 30 mA current and 40 kV. The X-ray diffractometer slit parameters were divergent of 1 deg, scatter of 1 deg and receiving of 0.3 mm. The measuring conditions were continuous scan mode, 3 deg/min scan speed, and 0.02 (deg) sampling pitch with presenting time of 0.4 s.

#### 2.4.10 Total phenolic and flavonoid content of pectin

The sample preparation for determining the total flavonoids and phenolic compounds was carried out following the procedure reported by Shraim, Ahmed [35]. First, 2 g of pectin was dissolved in 40 mL, and then its residue was extracted again using 20 mL of methanol. The two extracts were then blended. After that, the dissolved solids in the extract were dried at 40 °C using rotary evaporator, and a concentration of 50 mg/mL was prepared by re-dissolving the recovered solid in methanol. To determine the total flavonoid and phenolic content, standard concentrations of 20, 40, 60, 80, and 100 μg/mL were used. In this analysis, Quercetin was used as the standard for flavonoid content, while Gallic acid was used as the standard for phenolic content determination. The total flavonoid content (TFC) of coffee pulp pectin was determined using an AlCl_3_ assay as described by Shraim, Ahmed [35]. First, 2.0 mL of methanol, 500 μL of the sample solution (pectin or quercetin), and 200 μL of AlCl_3_ solution (10 % w/v) were mixed. Next, 200 μL of CH_3_COONa solution (1800 mg/mL) and 2000 μL of methanol were added and shaken. The resulting mixture was then placed in a dark place for 30 min, and absorbance was measured at 415 nm using a UV–Vis Spectrophotometer (V-770EX, Japan). The total phenolic compound was determined using the Folin-Ciocalteu (FC) Colourimetric analysis as described by Phuyal, Jha [36]. A 1000 μL of the sample (coffee pulp pectin or Gallic acid solution), 5000 μL of FC reagent (10 % v/v), and 4000 μL of Na_2_CO_3_ (7 %) were mixed and incubated for 30 min at 40 °C in an oven dryer and the absorbance was measured at 760 nm using a UV–Vis Spectrophotometer (V-770EX, Japan). Finally, the concentration of total flavonoid and phenolic content was calculated using equation (7) based on their respective calibration curve.

(7) $$A=c\frac{V}{m}$$

where A = total phenol or total flavonoid content (for flavonoid it is expressed in μg QE/mg while it is expressed as μg GAE/mg for total phenol), c = concentration from calibration curve, V = Volume and m = mass of the extract used.

#### 2.4.11 Antioxidant activity of pectin

The method reported by Wathoni, Shan [37], with some modification, was used to measure the antioxidant activity, using ascorbic acid as a reference. First, ethanol was used as a solvent to make pectin stock (3200 μg/mL) and DPPH (40 μg/mL) solution. Then, pectin stock solution was used to prepare solutions with 200, 400, 800, 1600, and 3200 μg/mL concentrations. Ascorbic acid was also produced at 2, 4, 8, 16, and 32 μg/mL solution concentrations. Then, pectin and ascorbic acid solutions (4 mL each) reacted with 6 mL of DPPH solution and put for 30 min. After that, absorbance was measured at 517 nm by UV–Vis Spectrophotometer (V-770EX, Japan) using ethanol (blank) and DPPH (control). Finally, IC_50_ level was calculated from the linear regression curve using equation (8).

(8) $$I\left(\%\right)=\left(1-\frac{\text{As}}{\text{Ab}}\right)\times 100$$

where, *I* is inhibition, *As* is absorbance of sample and *Ab* is blank absorption.

### 2.5 Experimental design and statistical analysis

A Box-Behnken Design (BBD) was employed to design and optimize pectin extraction conditions. BBD possesses good symmetry, produces minimal experimental runs and deliver maximal information [38]. The experiments involved four factors with three levels and total of thirty runs of which six are center points. The independent process variables for experiments were temperature (80–100 °C), time (45–75 min), SLR (1:20–1:35) and pH (1.5–2.5) while the response (dependent variable) was pectin yield.

(9) $$Y=a_{0}+\sum_{i=1}^{i}\left(a_{i}X_{i}\right)+\sum_{ij=1}^{ij}\left(a_{ij}X_{i}X_{j}\right)+\sum_{i=1}^{i}\left(a_{ii}X^{2}\right)$$

where, Y: yield, *a*_o_: constant, *a*_ij_: coefficients and X: factor.

Experiments were statistically analyzed using Design Expert 13 software to find the suitable model for the pectin yield as a function of the above variables. The statistical significance of independent parameters on pectin yield was analyzed through ANOVA and was significant if p < 0.05. Based on the optimal point obtained by the model equation, the experiment was triplicated and compared with the value obtained by the model. At the optimal point degree of esterification, acetyl value, equivalent weight, methoxyl content, ash content, and moisture content were determined by conducting experiments in triplicate.

## 3 Results and discussion

### 3.1 Optimization of pectin yield

#### 3.1.1 Model fitting and statistical analysis

BBD was used to optimize the pectin extraction process from the Harar coffee pulp using four independent factors (temperature, time, SLR, and pH) and six center points. The predicted R^2^: 0.9088, experimental R^2^: 0.9783 and adjusted R^2^: 0.958 (Table 2). These magnitudes of coefficients can give a reasonable prediction that represents the extraction conditions for pectin yield [39]. Furthermore, the ANOVA results on the yield of pectin (Table 2) has a model with p-value of less than 0.05 (significant), a lack of fit of 0.5943 (not significant), and a desirability of 0.984. These results showed that the model was adequate to predict pectin yield [40,41]. The modeling equation that was generated from the analysis was a second-order equation with respect to yield (equation (10)). Similarly, previous works also used quadratic model to optimize pectin extraction from coffee pulp [42,43].

(10) $$\text{Yield}=48.4344\;-\;1.382A+0.2964B+91.5946C\;-\;5.3977D\;-\;0.0007\text{AB}+5.1402\text{AC}\;-\;0.005\text{AD}\;-\;4.6729\text{BC}+0.0433\text{BD}+214.9533\text{CD}+0.0070A^{2}\;-\;0.0015B^{2}\;-\;6186.8577C^{2}\;-\;0.9333D^{2}$$

where, A is temperature, B is time, C is SLR (solid to liquid ratio) and D is pH.

**Fig. 1:**
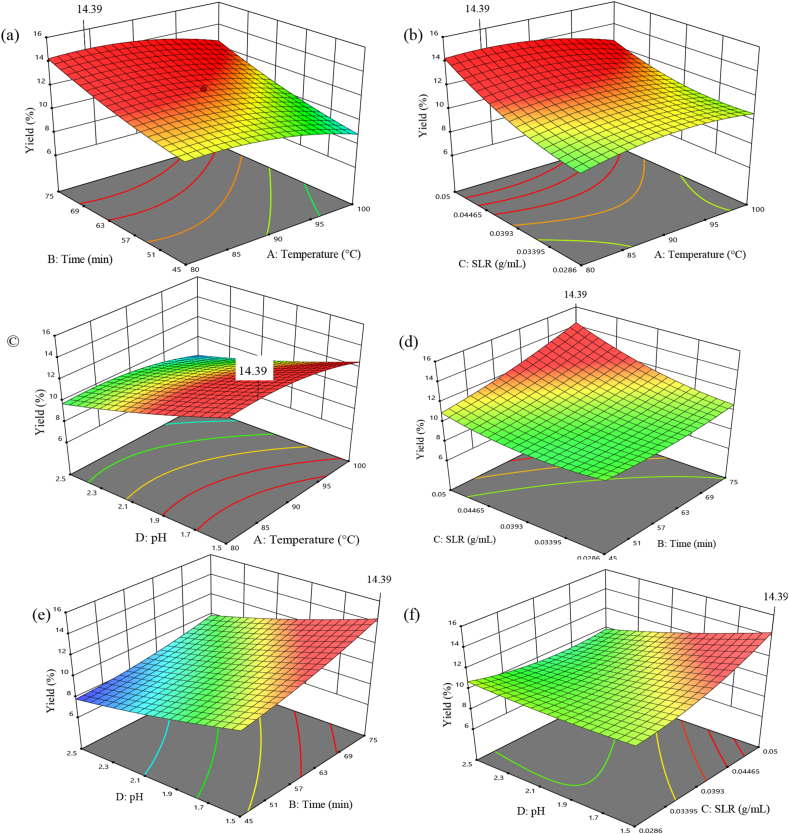
The optimal value and effects of the independent parameters' interactions (a) temperature - time; b) temperature - SLR; c) temperature - pH; d) time - SLR; e) time - pH; f) SLR - pH) effects on the coffee pulp pectin yield.

The yields of pectin from coffee pulp varied from 7.9 to 12.2 % (Table 1). The effects of main and interaction effects of the independent variables on the yield were indicated in equation (9) and Table 2. The higher coefficients imply that the impact of the independent variable on the responses is high. Therefore, the interaction effect of AC (Fig. 1b), BC (Fig. 1d) and CD (Fig. 1f) were more significant on the pectin yield. On the other hand, the interaction effects of AB (Fig. 1a), and AD (Fig. 1c) were smaller; hence, they have only considerable effects on the yields. The yield increased as temperature, time and SLR of extraction increased and pH decreased. This increment could be due to the hydrolyzing and mass transferring of pectin [41,44] from alcohol insoluble coffee pulp residue to the solvent.

**Table 1:** Experimental Design based Box-Behnken parameters and associated yields.

Run	Factors			Responses		
	A:Temperature (°C)	B: Time (min)	C: SLR	D: pH	Experimental yield	Predicted yield
1	90	60	1:27.5	2.0	9.7	9.6
2	80	60	1:27.5	2.5	8.9	8.7
3	90	60	1:35.0	2.5	11.2	11.3
4	90	60	1:27.5	2.0	9.6	9.6
5	90	60	1:27.5	2.0	9.3	9.6
6	80	75	1:27.5	2.0	10.2	10.2
7	80	60	1:27.5	1.5	10.4	10.3
8	90	60	1:20.0	2.5	8.4	8.3
9	90	45	1:27.5	2.5	8.9	9.1
10	90	60	1:20.0	1.5	12.2	12.0
11	100	60	1:27.5	1.5	9.3	9.5
12	90	45	1:35.0	2.0	11.3	11.1
13	100	60	1:20.0	2.0	8.3	8.3
14	90	75	1:35.0	2.0	10.8	10.9
15	90	60	1:27.5	2.0	9.8	9.6
16	90	60	1:27.5	2.0	9.8	9.6
17	90	75	1:20.0	2.0	11.5	11.7
18	90	45	1:20.0	2.0	9.0	8.9
19	80	60	1:35.0	2.0	9.7	9.8
20	90	75	1:27.5	2.5	9.9	9.8
21	100	75	1:27.5	2.0	9.7	9.6
22	90	75	1:27.5	1.5	12.0	11.9
23	100	60	1:35.0	2.0	10.3	10.1
24	100	60	1:27.5	2.5	7.9	8.0
25	80	60	1:20.0	2.0	9.9	10.2
26	90	45	1:27.5	1.5	9.7	9.9
27	90	60	1:27.5	2	9.3	9.6
28	100	45	1:27.5	2	8.1	8.1
29	80	45	1:27.5	2	9	9.0
30	90	60	1:35	1.5	10.4	10.5

**Table 2:** ANOVA quadratic model for yield.

Source	Sum of Squares	df	Mean Square	F-value	p-value	
Model	33.54	14	2.40	48.29	<0.0001	Significant
A-Temperature	1.69	1	1.69	34.01	<0.0001	
B-Time	5.47	1	5.47	110.21	<0.0001	
C-SLR	1.61	1	1.61	32.52	<0.0001	
D-pH	6.45	1	6.45	130.08	<0.0001	
AB	0.0400	1	0.0400	0.8063	0.3834	
AC	1.21	1	1.21	24.39	0.0002	
AD	0.0025	1	0.0025	0.0504	0.8254	
BC	2.25	1	2.25	45.35	<0.0001	
BD	0.4225	1	0.4225	8.52	0.0106	
CD	5.29	1	5.29	106.63	<0.0001	
A^2^	3.40	1	3.40	68.54	<0.0001	
B^2^	0.8201	1	0.8201	16.53	0.0010	
C^2^	3.44	1	3.44	69.35	<0.0001	
D^2^	0.3733	1	0.3733	7.53	0.0151	
Residual	0.7442	15	0.0496			
Lack of Fit	0.4758	10	0.0476	0.8866	0.5943	not significant

#### 3.1.2 Optimization of pectin extraction variables

The optimal conditions at which coffee pulp extracted to get maximum pectin yield was studied. It was observed that 84 °C, 75 min, a pH of 1.5, and SLR of 1:20 g/mL were the optimal extraction conditions. The highest yield from the model equation under optimal conditions was 14.39 %, while the mean result from actual experiments conducted in triplicate under optimal conditions was 13.7 ± 0.15 %. This demonstrated the model's capability for forecasting the pectin yield value when tested experimentally under optimum condition.

### 3.2 Physicochemical properties pectin

Table 3 displays the physicochemical characteristics of coffee pulp pectin under optimal extraction conditions. The detail physicochemical properties conducted for optimized pectin extraction were elaborated as follows.

**Table 3:** Properties and results of coffee pulp pectin extracted under the optimal conditions.

Properties	Results
**DA**	1.10 ± 0.05 %
**MeO**	7.9 ± 0.3 %
**DE**	78.5 ± 1.8 %
**EW**	1429 ± 54 g/mol
**AUA**	57.1 ± 0.9 %
**AC**	4.3 ± 0.9 %
**MC**	8.5 ± 1.5 %

where, DE is degree of esterification, MeO is methoxyl content, AUA is anhydrouronic acid, EW is equivalent weight, DA is degree of acetylation, AC is ash content & MC is moisture content.

#### 3.2.1 Acetyl values

Acetyl groups are situated at O-2 or O-3 on homogalacturonan (HG) backbone of pectin and it plays a emulsification role [45,46]. The acetyl value of extracted coffee pulp pectin was 1.10 % (Table 3). This value is similar with the Brazilian coffee pulp pectin reported by Reichembach and de Oliveira Petkowicz [19], but lower than pectin from Colombian coffee pulp (8.82 %) [23]. This value is also higher than reported acetyl values of apple peel (0.62 %) [29], but lower than apple pomace (1.21 %) [47]. As high acetyl levels indicate poor gelling capabilities of pectin, the low DA observed in coffee pulp pectin is advantageous for gel formation [48]. From these findings, it is possible to conclude that DA depends on sources and extraction methods.

#### 3.2.2 Degree of esterification

Pectin that was isolated from coffee pulp had DE values of 78.5 % (Table 3), indicating that it had a high level of esterification and may be utilized as an ingredient in jams and jellies [49]. Similar research on coffee pulp pectin by Reichembach and de Oliveira Petkowicz [19] revealed that its DE was 63.2 %, which is less than the present result. Contrarily, research by Chamyuang, Owatworakit [24] revealed that coffee pulp pectin had low methoxyl pectin levels. Due to increased de-esterification of polygalacturonic chains, pectin extracted under conditions of high temperature, prolonged extraction time, low pH, and low SLR has a low DE [41]. The moisture barrier performance of pectin films is influenced by the galacturonic acid content, DE, and acetylation, whereas the oxygen barrier performance is influenced by the galacturonic acid content and DE [50].

#### 3.2.3 Equivalent weight

Equivalent weight, which is dependent on the kind of plant, the quality of the raw material, the extraction technique, and the extraction process, measures the quantity of free galacturonic acid in the pectin [37,47]. It also indicates the amounts of methoxyl and carboxyl contents in the molecular structure of pectin. The higher the equivalent weight, the higher will be the methoxyl content and vice versa [51]. Pectin extracted at optimal extraction conditions from coffee pulp had an equivalent weight of 1429 g/mol (Table 3). This value is greater than the equivalent weight of the pectin extracted from coffee pulp by sodium hexamethaphosphate [51].

#### 3.2.4 Methoxyl content

The amount of methanol per 100 mol of galacturonic acid indicates the methoxyl content. It describes the quality, structure and texture of the pectin [52]. The methoxyl value for coffee pulp pectin extracted under optimized condition was 7.87 (Table 3) which indicates higher methoxyl pectin. The previous report by Hasanah, Setyowati [53] indicated the pectin from coffee pulp that was extracted using citric acid was a high methoxyl pectin. The authors also reported that methoxyl concentration influences pectin's solubility in water and its application, as it depends more on SLR than extraction time. The precipitation agents used during pectin recovery also affects the methoxyl content of pectin [54]. Kusrini, Wulandari [54] reported that pectin precipitated with methanol has higher methoxyl content than ethanol and propanol. But in this work, ethanol was used due to its lower toxicity and food-grade product compared to methanol.

#### 3.2.5 Anhydrouronic acid

The anhydrouronic acid (AUA) determines the purity of pectin. To be used as food additives and pharmaceutical products, the AUA of extracted pectin must not be less than 65 % [55,56]. The AUA contents of coffee pulp pectin extracted at an SLR of 1:20, a temperature of 83.9 °C, a time of 75 min, and a pH of 1.5 was 57.1 % (Table 3). It had to be noted that the AUA results in this work were for which ashes were not removed.

#### 3.2.6 Moisture and ash content

The moisture content of pectin (8.5 %) extracted based on optimized process conditions is comparable to the value (∼8 %) reported by Khamsucharit, Laohaphatanalert [57]. Minimal moisture level is required for safe storage as it hinders bacteria and pectinase growth [57]. The ash content represents the purity of the pectin. The lower the ash content, the higher the purity of the pectin [57]. The ash content of coffee pulp pectin (4.3 %) extracted at optimal conditions was lower than the values reported by Garcia, Arriola [22] (>5 %) from coffee mucilage), Wathoni, Shan [37] (7–10 % from mangoes) but higher than pectin from peels of banana (1.38–2.87 %), citrus peel (3.46 %) and pomace of apple (1.96 %) [57].

### 3.3 Morphological and structural properties

#### 3.3.1 FTIR analysis

**Fig. 2:**
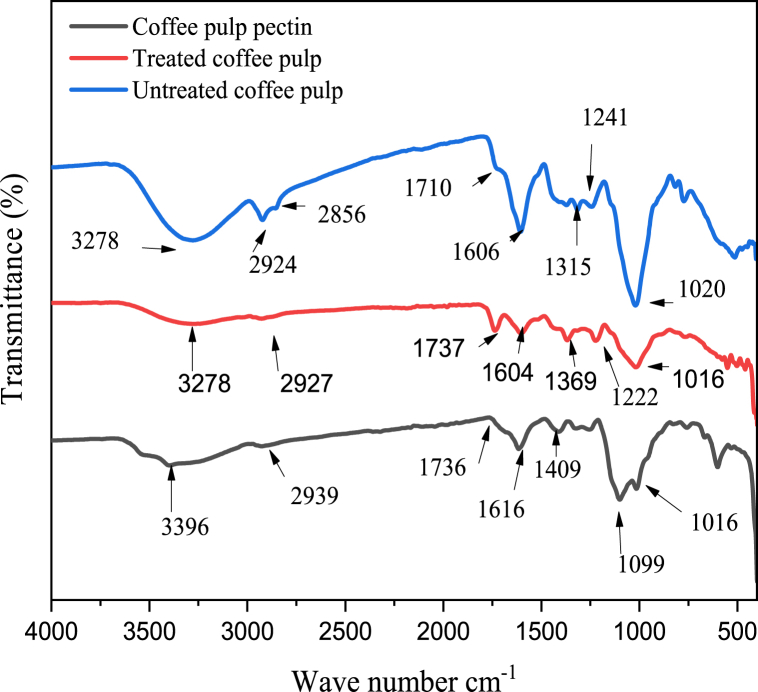
FTIR analysis of untreated coffee powder, alcohol treated coffee powder and pectin.

The FTIR analysis of the pectin extracted under optimal extraction conditions, raw coffee pulp powder, and coffee powder treated with alcohol are shown in Fig. 2. The coffee pulp pectin exhibited peaks at 3396, 2939, 1736, 1616, 1409, 1099, and 1016 cm^−1^. The untreated coffee pulp powder showed peaks at 3278, 2924, 2856, 1710, 1606, 1315, 1241, and 1020 cm^−1^, whereas the treated coffee pulp powder had peaks at 3278, 2927, 1737, 1604, 1369, 1222, and 1016 cm^−1^. The peaks at 3278 and 3396 cm^−1^ and 2924, 2927, and 2856 cm^−1^ are associated with –OH and (C–H)_x_ groups, respectively. The peaks at 1737, 1736, and 1710 cm^−1^ are assigned to esterified carboxyl group (-COOCH_3_), while the peaks at 1606, 1604, and 1616 cm^−1^ indicate stretching COO^−^ groups [20]. The peaks at 1315, 1020 and 1099 cm^−1^ correspond to the vibration of the C–O bond while peaks at 1241, 1222, and 1016 cm^−1^ represent the C–O–C linkage. Moreover, the spectral analysis revealed the presence of specific peaks at 1315 and 1099 cm^−1^, which correspond to the vibration of the C–O bond; 1409, 1369 and 2939 cm^−1^, the vibration of the C–H bond. FTIR peak intensity changes during preparation of coffee pulp pectin. The characteristic chemical shifts of each sample (pectin, treated and untreated coffee pulp powder) might be due to the effects of bond length as a result of electronegativity changes of the neighboring, such as hydrogen bonding [58]. Pectin had a hydroxyl peak at 3396 cm^−1^ wavenumber which is a higher wavenumber as compared to the treated and untreated coffee pulp powders wavenumber (3278 cm^−1^) due to inter- and intra-molecular hydrogen bonds between O–H, (C–H)_x_, C–O–C in glycoside complex [27,37]. During coffee pulp pectin extraction, cellulose, hemicellulose, and lignin are expected to be removed from coffee pulp pectin. As a result, coffee pulp pectin has a different FTIR structure from the coffee pulp powder.

#### 3.3.2 Morphological analysis using SEM

**Fig. 3:**
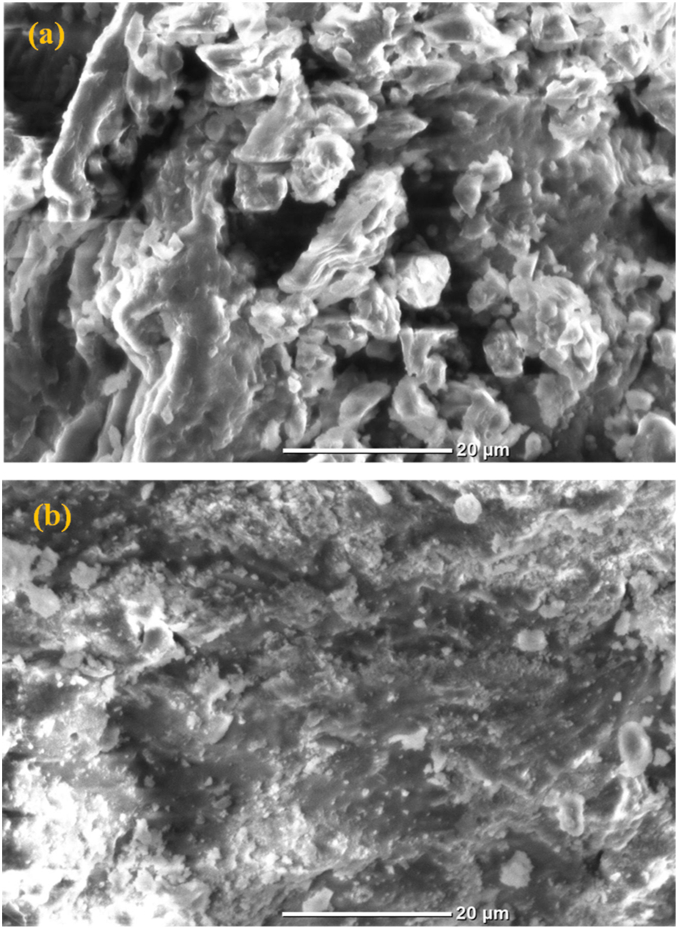
SEM images of coffee pulp powder and pectin. (a) Coffee pulp powder, (b) Coffee pulp pectin.

The morphology of the coffee pulp pectin (Fig. 3b) extracted under optimal conditions (temperature: 84 °C, extraction time: 75 min, solid-liquid ratio: 1:20, and pH: 1.5) and the coffee pulp powder (Fig. 3a) was analyzed using SEM. The surface of pectin appears smoother than that of the coffee pulp powder. However, coffee pulp powder showed wrinkled ruptures, roughness, and irregularity. These characteristics may be attributed to the presence of sugar, proteins, fibre, and macromolecules [37]. The presence of these components in the pectin reduce its quality [19]. Wathoni, Shan [37] reported that the extracted pectin exhibits a rougher and more fragmented surface compared to commercial pectin, possibly due to the modification of commercial pectin by sugar.

#### 3.3.3 XRD

**Fig. 4:**
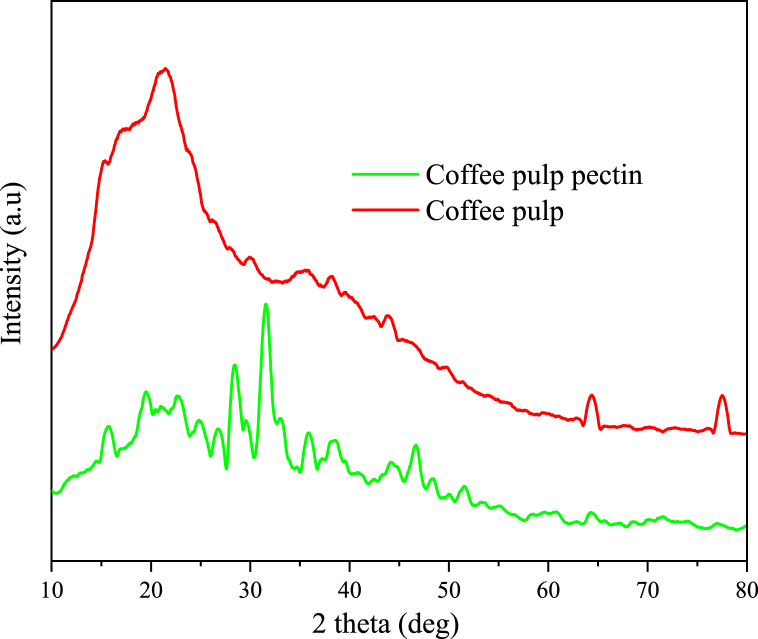
XRD of coffee pulp powder and pectin.

Fig. 4 shows the XRD patterns of coffee pulp pectin extracted under optimal condition (temperature: 84 °C, extraction time: 75 min, solid-liquid ratio: 1:20, and pH: 1.5) and coffee pulp powder. The pattern revealed that the coffee pulp powder is more amorphous compared to the coffee pulp pectin extracted under optimal conditions. The coffee pulp pectin exhibited sharp peaks at (2θ) values of 15.72, 19.52, 20.90, 22.94, 25.02, 26.88, 28.44, 29.59, 31.59, 32.94, 35.88, 38.32, 44.5, 46.66, 48.2, and 51.44, indicating its crystallinity. The pectin does not exhibit visible peaks at 64.38 and 77.5, which may be associated with the removal of cellulose, hemicellulose, and lignin. On the other hand, the coffee pulp powder displayed peaks at 14.96, 16.95, 20.90, 21.02, 21.92, 30.18, 38.22, 43.98, 64.38, and 77.50. The peaks in the coffee pulp powder are less intense than those in the pectin, suggesting that the coffee pulp pectin has a higher degree of crystallinity compared to the coffee pulp powder. This crystalline structure is crucial in determining its thermal and other related properties. Moreover, both amorphous and crystalline structures are present, similar to the XRD results of sour orange pectin [27].

### 3.4 Total phenolic and flavonoid content

The total phenolic content in pectin was measured to be 26.7 μg GAE/mg of sample. This result was similar to the result reported by Manasa, Padmanabhan [1], who studied coffee pulp pectin using HPLC. However, this value is higher than the values reported for apple pectin, which were described in terms of Ferulic acid equivalence [59]. On the other hand, the total flavonoid content was determined to be 0.845 μg QE/mg, which indicated it is lower than total phenols. These findings suggest that the product may have antioxidant properties, which can be attributed to the presence of polyphenols in coffee pulp pectin.

### 3.5 Antioxidant activity of pectin

**Fig. 5:**
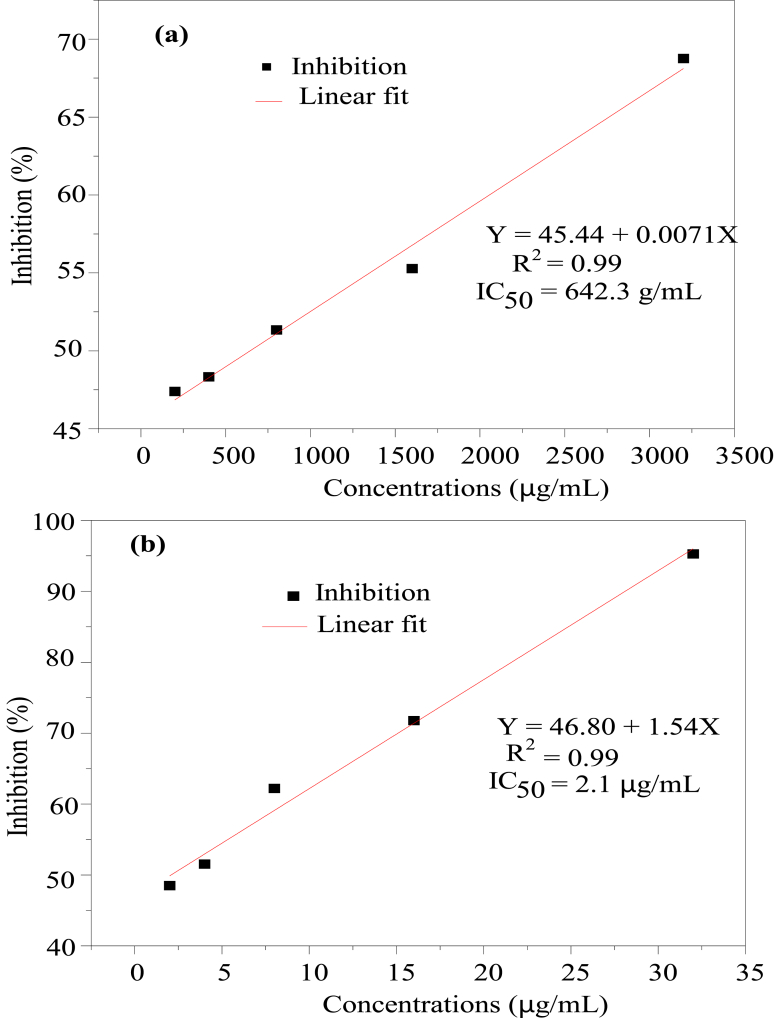
Antioxidant property of coffee pulp pectin and ascorbic acid. (a) Coffee pulp pectin, (b) ascorbic acid.

The antioxidant activity of coffee pulp pectin was assessed using DPPH radical scavenging assay. When antioxidant components scavenge DPPH free radicals, colour of the samples changes from purple to yellow due to the formation of DPPH-H [37]; hence, samples give different colour depending on its concentration. The hydroxyl structure and phenol compounds of pectin help pectin to have antioxidant properties [60]. The antioxidant activities of pectin and ascorbic acid were shown in Fig. 5. The antioxidant activity of coffee pulp pectin at different concentrations of 200, 400, 800, 1600 and 3200 μg/mL (Fig. 5a) were determined and compared against various ascorbic acid concentration levels of 2, 4, 8, 16 and 32 μg/mL (Fig. 5b). The result indicated that as both concentration of pectin and ascorbic acid increased their antioxidant property also increased. The IC_50_ was 642.31 and 2.1 μg/mL for coffee pulp pectin and ascorbic acid, respectively. It shows that although coffee pulp pectin contains antioxidant properties, they are substantially less potent than ascorbic acid. Wathoni, Shan [37] reported that IC_50_ of pectin from Indonesian mangosteen rind extract has moderate antioxidant (161.93 ± 31.57 μg/mL). Pectin properties such as high total phenolic compounds contributed to higher antioxidant properties [27].

## 4 Conclusion

Pectin was extracted from the coffee pulp using sulfuric acid. The pectin yields ranged from 7.9 to 12.2 %. The optimal conditions for isolating the coffee pulp pectin with the best yield were determined: temperature (84 °C), time (75 min), pH (1.5), and SLR (1:20). Under these conditions, the coffee pulp pectin yield was 13.7 ± 0.15 % and being near the expected value of 14.39 %. The properties of coffee pulp pectin extracted under optimal condition showed that it has a high methoxyl content, an acceptable range of degree of acetylation, and higher equivalent weight while its anhydrouronic acid near to the acceptable level. The FTIR analysis showed that the distinctive bands in commercial pectin were also present in the extracted coffee pulp pectin. The XRD pattern showed that pectin has an amorphous structure, and the SEM study also revealed morphological variations between the raw material and pectin. The study using DPPH radical scavenging assay also revealed that there are considerable antioxidants in extracted coffee pulp pectin. Therefore, it can be concluded that acceptable quality pectin can be obtained from coffee pulp with potential applications in the food and pharmaceutical industries. This study, on the other hand, may have drawbacks. The use of DPPH alone to test antioxidants may not produce reliable antioxidant characteristics; another drawback is that optimization is done for yield rather than other pectin qualities such as DE and DA.

## Funding statement

This work was supported by the Addis Ababa Science and Technology internal research grant (IGP 002/2023).

## Data availability

Data will be made available on request.

## CRediT authorship contribution statement

**Girma Biratu:** Formal analysis, Conceptualization. **Henock Woldemichael Woldemariam:** Writing – review & editing. **Girma Gonfa:** Writing – review & editing, Formal analysis, Conceptualization.

## Declaration of competing interest

The authors declare that they have no known competing financial interests or personal relationships that could have appeared to influence the work reported in this paper.
